# Associations of Digital Measurements: Analysis of Orthopantomography Versus Lateral Cephalograms for Evaluation of Facial Asymmetry

**DOI:** 10.3390/jcm14041296

**Published:** 2025-02-15

**Authors:** Andra-Alexandra Stăncioiu, Alexandru Cătălin Motofelea, Anca Adriana Hușanu, Lorena Vasica, Riham Nagib, Adelina Popa, Camelia Szuhanek

**Affiliations:** 1 Orthodontic Research Center ORTHO-CENTER, Discipline of Orthodontics I, Faculty of Dental Medicine, “Victor Babes” University of Medicine and Pharmacy Timisoara, 9 No., Revolutiei Bv., 300041 Timisoara, Romania; andra.stancioiu@umft.ro (A.-A.S.); husanu.anca@umft.ro (A.A.H.); nagib.riham@umft.ro (R.N.); popa.adelina@umft.ro (A.P.); cameliaszuhanek@umft.ro (C.S.); 2 Dental Clinic Arad, Vicențiu Babeș Sq., 310029 Arad, Romania; lorenavasica@yahoo.com; 3 Center for Molecular Research in Nephrology and Vascular Disease, Discipline of Nephrology, Department VII/Internal Medicine II, Faculty of Medicine, “Victor Babeș” University of Medicine and Pharmacy, 300041 Timișoara, Romania

**Keywords:** ANB, artificial intelligence, dentistry, digital analysis, FMA, gonial angle, mandibular body length, orthodontics, ramus height, WebCeph

## Abstract

**Background/Objectives**: This study aimed to compare the associations of digital measurements obtained from orthopantomographies (OPGs) or panoramic radiographs and lateral cephalograms in evaluating facial asymmetry in patients with different skeletal classes. The sample consisted of 128 Romanian patients (67 females, 61 males) who sought orthodontic treatment. These measurements are an essential diagnostic tool for evaluating facial asymmetry in order to treat them. **Methods**: Lateral cephalograms and OPGs were obtained for each patient, and digital tracing was performed using the WebCeph program. Angular measurements (ANB, FMA, gonial angles) and linear measurements (ramus height, mandibular body length) were assessed on both imaging modalities. **Results**: Strong positive correlations were found between the gonial angle and ramus height measurements obtained from lateral cephalograms and OPGs (rs range: 0.800–0.946; *p* < 0.001). However, the mandibular body length showed weaker correlations between the two methods. Significant sex differences were observed, with males exhibiting larger craniofacial measurements compared to females (*p* < 0.05). The study population was quite young, as seen by the cohort’s median age of 21 years and interquartile range (IQR) of 16 to 29 years. Lateral Ceph: the FMA angle median value of 22° (IQR: 17–25), gonial angle median of 121° (IQR: 116–127), mandibular ramus height median value of 44 mm (IQR: 41–48 mm), and mandibular body length median value of 70 mm (IQR of 65 to 76 mm). OPG: gonial angles on the right and left sides yield medians of 121° (IQR: 116–127) and 122° (IQR: 117–127); the mandibular ramus height on the right and left sides shows medians of 44.0 mm (IQR: 40.0–47.0 mm) and 43 mm (IQR: 40–48 mm); and the mandibular body on the right side presents a median of 71 mm (IQR: 67–76 mm) and the left side has a median of 71 mm (IQR: 67–75 mm). **Conclusions**: The findings suggest that OPGs can be reliably used to measure the gonial angle and ramus height, providing results comparable to lateral cephalograms. However, caution should be exercised when predicting horizontal measurements from OPGs. The standardization of the OPG recording process and further research with larger sample sizes are required to establish standard panoramic norms for OPG parameters in the assessment of facial asymmetry.

## 1. Introduction

The success of orthodontic therapy and dental care depends on a thorough patient assessment. A comprehensive evaluation of occlusion, soft tissue connections, and the skeletal form is required for diagnosis and therapy planning, typically involving radiographs, dental casts, clinical examinations, and patient photos [1]. Among these, panoramic radiographs are an essential tool for orthodontic diagnosis and treatment planning, providing a broad view of the mandibular and maxillary arches, temporomandibular joints, and supporting tissues. Introduced by Professor Yrjo Paatero in 1961, panoramic radiographs are valued for their non-invasiveness, lower radiation exposure, affordability, and ability to display the entire dentition and surrounding anatomy [2].

Since Levandoski first analyzed panoramic radiographs in 1991, research on their quantitative accuracy has been limited, partly due to the inherent variability in magnification [3]. Despite these limitations, panoramic radiographs remain integral to orthodontic and dental diagnosis, aiding in the detection of mandibular asymmetry, dental anomalies, tooth eruption patterns, bone pathology, and cysts or tumors. They also play a critical role in monitoring orthodontic therapy, as highlighted by Graber’s 1967 recommendation for their routine use [4].

Cephalometric radiography has been a cornerstone of orthodontic diagnosis and treatment planning since its introduction by Broadbent in 1931 [5]. This imaging technique enables the precise assessment of dentofacial proportions, identification of skeletal discrepancies, and evaluation of growth- and treatment-related changes [6]. Cephalometric analysis is particularly critical in cases of skeletal malocclusion, where treatment outcomes depend on an accurate understanding of craniofacial relationships [7].

With advancements in imaging technology, traditional manual cephalometric tracing has been largely replaced by digital methods. The transition to digital radiography has improved diagnostic accuracy by reducing human error, increasing reproducibility, and allowing for rapid image acquisition and enhancement [8]. Early digital systems relied on digitizer pads and scanners to convert analog radiographs, but modern direct digital imaging eliminates many technique-sensitive processes. Digital cephalometry not only minimizes radiation exposure, but also enables automated measurements, making it an essential tool in contemporary orthodontic practice [9].

Facial asymmetry, while common to some degree in the general population, can have significant functional and esthetic implications when severe. Its etiology includes congenital, acquired, and developmental factors [10,11,12]. An accurate diagnosis often involves supplemental imaging, intraoral and extraoral examinations, and advanced radiographic techniques like orthopantomograms (OPGs) and lateral cephalograms. OPGs, in particular, allow the separate evaluation of right and left structures, mitigating the superimposition challenges of lateral cephalograms. However, questions remain about the validity of certain cephalometric measurements taken on OPGs, warranting further investigation [13,14].

AI-based algorithms are increasingly widely used in technology, and the technology is used in many facets of daily life. Recent advancements in computers have made it possible to apply AI algorithms for both simple and complex tasks, which have promise for a number of healthcare domains [7].

Recent advancements in artificial intelligence (AI) have revolutionized medical imaging, including dental radiographs. AI-powered algorithms now assist in landmark identification and measurement analysis, potentially reducing human error and enabling fewer experienced practitioners to achieve consistent results. Despite its promise, AI implementation in orthodontics often requires the manual input of measurements, introducing the possibility of missing or incomplete data [15].

The orthopantomogram (OPG) is a cost-effective imaging technique for assessing the mandibular condyle, detecting pathognomonic changes such as condylar inclination, flattening, and bone loss. While OPGs provide broad anatomical insights, lateral cephalograms remain the gold standard for evaluating skeletal discrepancies, including open bite, overjet, and facial height variations [16].

WebCeph, an AI-powered orthodontic platform, enhances image archiving, cephalometric tracing, and treatment simulation, improving diagnostic precision and reproducibility. As AI continues to evolve, its role in validating OPG-derived cephalometric measurements warrants further research, particularly for the asymmetry assessment and skeletal morphology analysis. Integrating AI with OPG imaging could enhance orthodontic diagnostics, offering a viable alternative or complement to lateral cephalograms.

The current study aims to evaluate the associations of digital orthopantomography imaging in determining gonial angles, the ramus height, and mandibular body length compared to lateral cephalograms in patients presenting with various skeletal classes. By addressing the limitations of traditional methods and exploring the integration of modern technologies, this research seeks to enhance the diagnostic precision and improve orthodontic treatment outcomes.

## 2. Materials and Methods

### 2.1. Ethical Considerations

This study was conducted on patients enrolled in the Discipline of Orthodontics I, Faculty of Dental Medicine, “Victor Babeș” University of Medicine and Pharmacy in Timișoara. Written informed consent was obtained from all participants, and ethical approval was granted by the Scientific Research Ethics Committee of the “Victor Babeș” University of Medicine and Pharmacy, Timișoara, Romania (CECS number 04/26 January 2024).

### 2.2. Study Design and Participants

This cross-sectional study included 128 Romanian patients who sought orthodontic treatment at the university’s clinic. Each patient underwent a comprehensive clinical assessment, including radiographs, pre-treatment study models, and clinical photographs. Inclusion criteria comprised individuals over a specified age, those with good oral hygiene, Romanian nationals, and patients willing to participate in this study. Exclusion criteria included prior orthodontic or orthognathic surgical interventions, history of craniofacial clefts, use of hormonal medications, endocrine disorders, neurological conditions, or maxillofacial trauma.

### 2.3. Procedure Methodology

#### 2.3.1. Protocol and Measurements for Digital Lateral Cephalometry and Orthopantomography

All patients’ lateral cephalograms were obtained with their heads in a natural position as a reference [17]. Orthopantomograms (OPGs), or panoramic radiographs, were also performed with the patient’s head in a natural position, ensuring that each participant’s visual axis was parallel to the floor and directed straight ahead [18].

During an OPG, the patient remains motionless (either standing or sitting) while the film and X-ray source rotate around the head. The X-ray source moves from one side of the jaw to the other and then around the front, while the film rotates in the opposite direction. Patients must stay still for a few seconds. Technical aspects of the panoramic image—such as paused breathing (varying by department), focal points, and laser lights—depend on the equipment vendor. However, common guidelines include aligning the center midsagittal laser light, the axial laser at the IOML, and a laser directed laterally at the lateral incisor. Before imaging, patients must remove any glasses, jewelry, dentures, and hearing aids. The patient is positioned standing or sitting fully upright, with the head on a chin rest, a radiolucent bite block in place, and the tongue against the hard palate to minimize distortion. Proper positioning is essential for obtaining a crisp, precise, and distortion-free image. Notably, 60–96% of OPGs have positioning errors, and 5–33% of these images become uninterpretable. The most frequent mistake is failing to keep the tongue against the hard palate. Understanding such errors and their impact on image quality is crucial for accurate interpretation [19].

OPGs are commonly prescribed as a screening tool, providing a complete view of the teeth and jaws. The radiation dose is approximately 0.014 mSv, which is lower than the 0.02 mSv dose of a standard chest X-ray. Being an extraoral radiograph, OPGs are especially useful when a patient has difficulty opening their mouth or exhibits a prominent gag reflex, preventing the use of intraoral films.

During exposure, the X-ray generator head and the detection unit circle are around the patient’s head. This movement focuses on the teeth and mandible, but can result in artifacts if the patient moves. Due to the bite block used to position the patient in the correct focal trough, the mandible is often recorded in a slightly protruded position, placing the condylar head anterior to its rest position in the glenoid fossa. The cervical spine may obscure anterior teeth through superimposition, and the occipital bone may appear over the ramus on the opposite side [20].

Despite improvements such as positioning devices and image registration, panoramic radiography still presents limitations and challenges. Certain patients—such as those with anatomical or pathological variations, those who are uncooperative, or those with movement disorders—may not be ideal candidates for obtaining a satisfactory diagnostic panoramic image. Artifacts and errors can occur at any point during the panoramic procedure. Individuals who are asleep, very young, or unable to keep their cervical spine upright cannot have panoramic radiographs taken. Furthermore, some jurisdictions prohibit panoramic radiography for children under five years old. Tremors, jerks, tics, or seizures may lead to motion artifacts or require stopping the procedure. Machines with motion compensation are necessary for patients with conditions like Parkinson’s disease. Cognitive impairments or dementia can also complicate the process, as patients may struggle to understand or follow positioning instructions.

In cases of severe Class II or Class III malocclusion, placing upper and lower incisors in the bite block grooves can be difficult. Patients with advanced periodontal disease and tooth mobility may be unable to bite properly in the bite piece’s groove without causing tooth tilt when the mouth closes. It may be necessary to use a cotton roll and chin support to maintain an appropriate position. Patients with lockjaw, lip or tongue injuries, or post-trauma swelling may also need a cotton roll for chin support [18].

During radiography exposure, patients were placed upright, shoulders relaxed, back straight, feet closed, head straight over the chin rest, biting on a bite block, tongue against the hard palate, and head with the median sagittal plane perpendicular to the ground and the Frankfort plane parallel to the floor. Patients were positioned in a natural head position for cephalometric radiographs, with the lips in relaxed contact, the teeth in central occlusion, and the gaze straight ahead. The patients were positioned with the Frankfort horizontal plane parallel to the floor and the external auditory meatus under mild pressure from the cephalostat’s ear rods. The radiography images’ crispness and quality were assessed.

Using a PaX-i3D Green (Vatech, Hwaseong, Republic of Korea) device, qualified radiography technicians took orthopantomographies (OPGs) or panoramic radiographs and lateral cephalograms (Tube: 50–100 kVp (1 kV step)/4–16 mA (0.1 mA step). Patient Position: Standing Wheelchair Accessible. Scan Time: Orthomantomography: 10.1 s (normal) and lateral ceph: 3.9 s.

WebCeph is an A.I.-web-based orthodontic and orthognathic platform.

We used the WebCeph program (version 2.0.0) for digital tracing. Before being imported into the WebCeph, each digital image from a lateral cephalometric radiograph and panoramic radiographs was downloaded and saved to a Lenovo IdeaPad 5 Pro computer (Lenovo Group Ltd., Arad, Romania). Once the radiological images were uploaded to the WebCeph application, the system instantly recognized each anatomical site for lateral cephalometric radiographs. Both the automated and semi-automatic WebCeph options were used to identify landmarks on the digital photos, also for lateral cephalometric radiographs. The size of the images for the lateral cephalogram was 2430 × 3016, with a width of 2430 pixels, a height of 3016 pixels, a horizontal and vertical resolution of 96 dpi, and a bit depth of 24, and for the panoramic radiographs, it was 3070 × 2376, with a width of 3070 pixels, a height of 2376 pixels, a horizontal and vertical resolution of 96 dpi, and a bit depth of 24.

Several researchers have used WebCeph for landmark detection [15,21,22].

A calibration of 10 mm was used for the lateral cephalograms and 65 px for the linear measurements that were performed on the panoramic radiographs. We used WebCeph to import the digital radiographs that were saved as JPEG files. We used a 14” screen to perform the automated analysis. The WebCeph^®^ (Korean Intellectual Property Office, Seoul, Republic of Korea, WebCeph, 2.0.0 AssembleCircle Corp, Pangyoyeok-ro, Bundang-gu, Seongnam-si, Gyeonggi-do, Republic of Korea) software program for digital tracing was used to perform the cephalometric analysis and measure panoramic radiographs. Both the Korean and US Intellectual Property Offices have granted patents to WebCeph’s AI inventions.

Before being imported into WebCeph, each digital image greyscale file was downloaded and stored on a Lenovo IdeaPad 5 Pro computer.

Three angular measurements were made on the lateral cephalograms: the ANB angle, the FMA angle (to classify the patients in their skeletal class), and the gonial angle; linear measurements such as the height of the mandibular ramus and the length of the mandibular body were also made. On the panoramic radiographs, we had two angular measurements, the gonial angles, and linear measurements such as the height of the ascending ramus—Sigmoid notch point–Antegonion (Snp-Ag)—on the right and left sides and the length of the mandibular body—Gonion–Mandibular Midpoint (Go-M)—the same for the right and left sides.

We used these parameters in this study because FMA and ANB show the vertical and sagittal skeletal growth pattern, and the gonial angle, mandibular ramus height, and mandibular body length because these parameters can be found in WebCeph and can be measured on OPG and on the lateral ceph.

Exclusion criteria in this study: patients who had had surgical orthodontic treatment in the past, patients who had had orthodontic treatment in the past of any type, patients with a history of craniofacial clefts, patients with neurological disorders, endocrine disorders, or hormonal medication, and jaw trauma facial. Inclusion criteria were patients over seven years old, patients who wanted to participate in this study, patients with good dental hygiene, patients from Romania, and patients who presented themselves for orthodontic treatment.

Measurements were taken based on these landmarks, and planes are presented in Table 1 below.

The ANB angle measures the difference between the mandible and maxilla in the sagittal plane. Skeletal class I, or 2°, is the normal value. Higher values indicate Skeletal class II. Skeletal class III corresponds to lower and even negative values.

Nasion is the most anterior point on the frontonasal suture in the medio-sagittal plane and corresponds to the root of the nose. A point represents the most posterior point on the anterior nasal spine’s curvature, situated below the SNA and in front of the root of the upper central incisor. The bone contour’s most posterior point lies between the SNA and Pr. B point is the most posterior point on the profile of the mandibular alveolar process, between Id and Pog—an anatomical, bony median point.

The FMA angle allows us to see the skeletal typology in a vertical orientation. The mandibular plane and the Frankfurt plane comprise this angle. The typical value is 25 ± 3°. The growth type is normodivergent when the value falls within normal bounds. The growth type is hyperdivergent when the value exceeds 28°. Hypodivergent growth occurs when the value is less than 22°.

Frankfurt Plane made up of Po-O (Porion–Orbital); Porion is the highest (external) point on the bony border of the external auditory canal—an anatomical point, bony, bilateral. Orbital is the lowest point on the lower edge of the bony orbit. Anatomical point: bone, bilateral.

Mandibular Plane is composed of Go-Me (Gonion-Menton), a tangent between the two sites of greatest convexity on the inferior border of the mandible that forms the lower border of the mandibular body. Gonion is the most posterior, lateral, and inferior point on the external face of the angle of the mandible. Menton is the lowest point on the chin symphysis [26].

The term “gonial angle” refers to the angle that forms when a tangent touches the posterior border of the ramus at two points: the angle region (Broca) and the condyle. It has also been used to describe the posterior inferior angle region of the mandible (Brodie). In humans, the gonial angle can range from 100 to 148 degrees. Its mean angle is lowest among early Caucasians, Australians, and American Indians, and it is highest among Caucasians, Chinese, Eskimos, and Negroes. The mean angle of females is 3 to 5 degrees greater than that of males across all racial groups. According to cross-sectional studies, the gonial angle grows larger from the earliest stages of embryogenesis until birth and then continuously shrinks from birth until old age. The rate of decline is approximately half as high until adulthood, and it only decreases by a few degrees from maturity to old age. The decline is greatest before the age of six. Complete tooth loss may cause the gonial angle to become more obtuse once more, reversing the normal aging changes [27].

The mean value of Gonial Angle is 122.81 ± 10.68. Ramus height (minus condyle and coronoid process): measured from point Snp to Ag point, the mean value is 46.22 ± 6.34, the separation between the antegonion point and the sigmoid notch point (without including the condyle and coronoid). Mandibular body length: measured from point Go to point M, the mean value is 88.52 ± 9.35, from the gonion point to the mandibular midpoint. Sigmoid notch point (SNP): the deepest point on the sigmoid/mandibular notch. Antegonion (Ag): the highest point of the concavity or notch on the ramus’ lower border where it connects to the mandibular body. Mandibular midpoint (M): by projecting the mental spine onto the lower mandibular boundary parallel to the ANS vertical plane, one can determine the mandibular midpoint (M) [25].

#### 2.3.2. Reliability Analysis

To ensure measurement reliability, intra-rater consistency was assessed. A single examiner performed all measurements, and a randomly selected 20% of the dataset was re-analyzed after a two-week interval. The intraclass correlation coefficient (ICC) was computed, with values greater than 0.75 considered indicative of good reliability.

**Figure 1:**
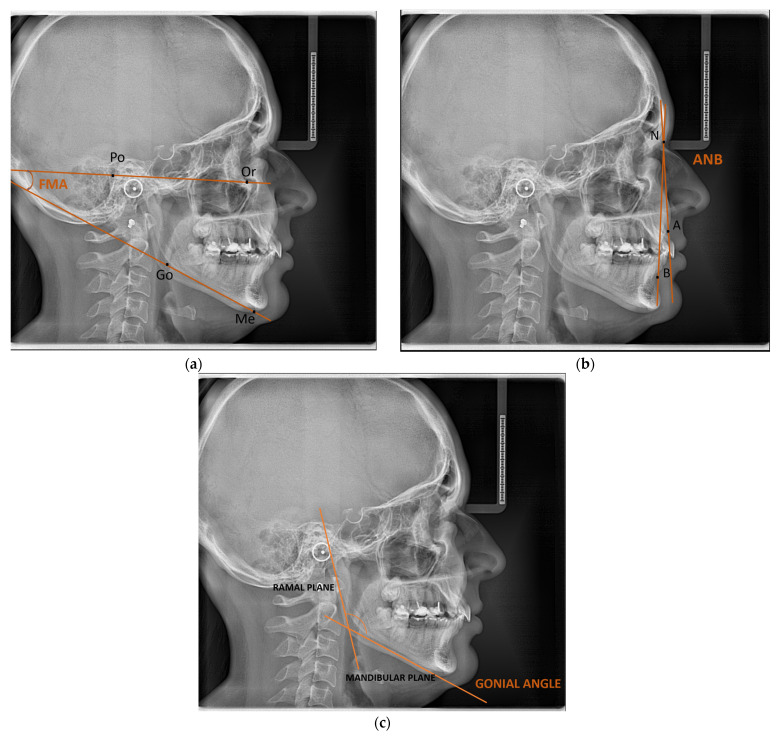
(**a**) ANB angle, (**b**) FMA angle, and (**c**) gonial angle.

In the same way that they would position a patient’s head in a cephalostat, radiologists positioned the skulls in three dimensions. The center axis of the skull should run parallel to the lateral film, which should appropriately cover the bilateral structures on the mandibular inferior border (Figure 1).

Figure 1 below shows the angle that we drew for this study. In Figure 1a, we have the FMA angle (FH plane and the mandibular plane), in Figure 1b, we have the ANB angle (Nasion–Apoint and the Nasion–Bpoint plane), and in Figure 1c, we have the gonial angle (the mandibular plane and the ramus plane’s built point of junction), all drawn on a lateral cephalogram.

**Figure 2:**
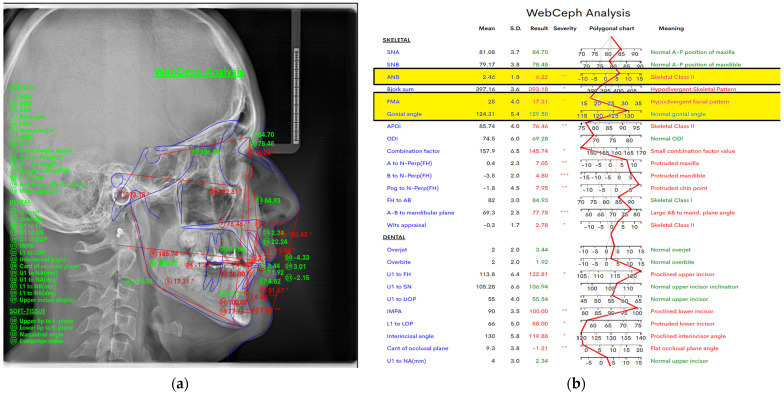
The WebCeph digital program in which we measured the ANB angle, the FMA angle, and the gonial angle. (**a**) Image-view mode by line analysis and image (**b**) by a chart.

Figure 2 below shows the angular measurements that were made in the WebCeph digital program. We measured the ANB angle, the FMA angle, and also measured the gonial angle. The gonial angle will be measured bilaterally in orthopantomography, as we will see below. In Figure 2a, we have represented the view mode line analysis, and in Figure 2b, we have represented the chart and the mean values, standard deviation, resultant values, severity (which is indicated by the symbol “*”; one “*” indicates the least degree of severity, and more “*” indicate a greater degree of severity), and polygonal charts, and their interpretations are all shown in detail.

**Figure 3:**
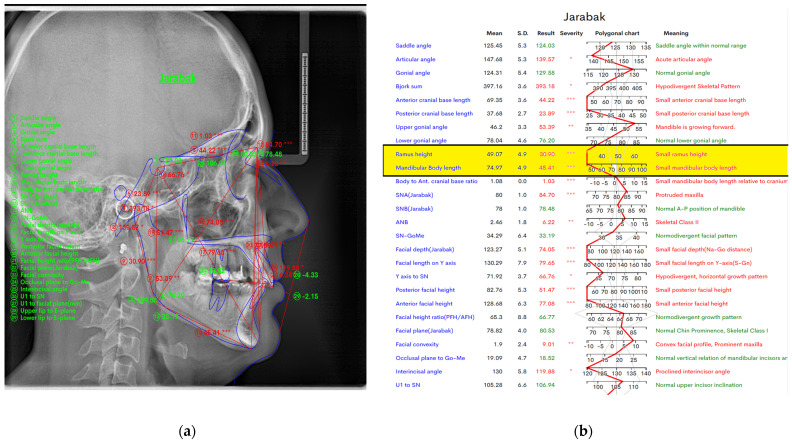
In the WebCeph digital program, we measured ramus height and mandibular body length (**a**) in figure-view mode by line analysis and image (**b**) by a chart.

In Figure 3 below, we also measured ramus height and mandibular body length. In view mode, in Figure 3a, line analysis is presented, and in Figure 3b, it is represented by a chart. A detailed display of the mean values, standard deviation, resultant values, severity (represented by the symbol “*”; one “*” denotes the least degree of severity, and more “*” denote a greater degree of severity), and polygonal charts, and their interpretations are provided.

**Figure 4:**
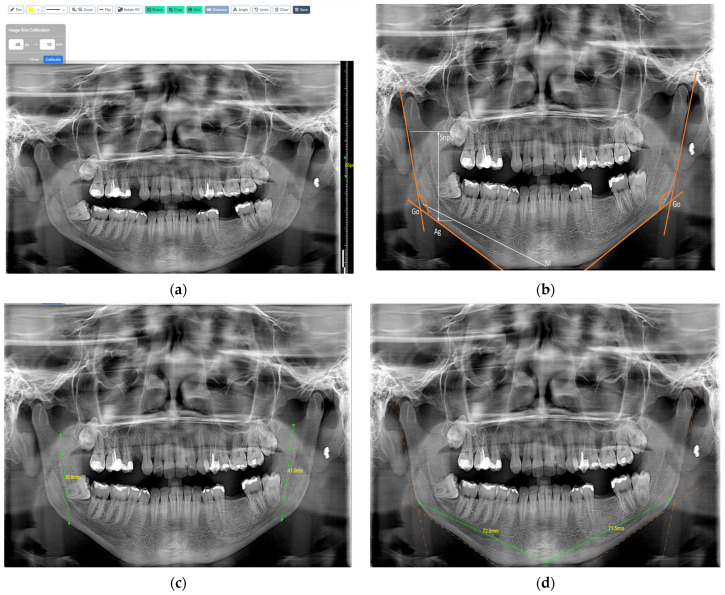
The WebCeph digital program in which image (**a**) is the calibration for the linear measurements performed, image (**b**) is right and left gonial angles and the linear measurements performed: ramus height and mandibular body length. (**c**) is ramus height and (**d**) is mandibular body length, all shown exactly as we drew.

Figure 4 follows in which, in Figure 4a, we showed how we calibrated the panoramic radiographs for the linear measurements at 65 pixels, taking the reference and the vertical ruler next to it. Since the technique used to obtain panoramic radiographs greatly distorts the horizontal length, it can be difficult to quantify the horizontal dimension accurately. Measurements taken vertically are generally more accurate than those taken horizontally because the vertical dimension is less susceptible to this kind of distortion. In general, panoramic radiographs are sufficiently distorted, and it is challenging to offer a uniform pixel conversion value since different radiography machines provide varying image sizes. In Figure 4b, we first drew right and left gonial angles, and after, we measured ramus height from Snp-Ag (sigmoid notch point and antegonion) and mandibular body length Go-M (mandibular midpoint and gonion). These measurements were performed bilaterally for both the right and left sides. In Figure 4c,d, we showed how we drew digitally.

**Figure 5:**
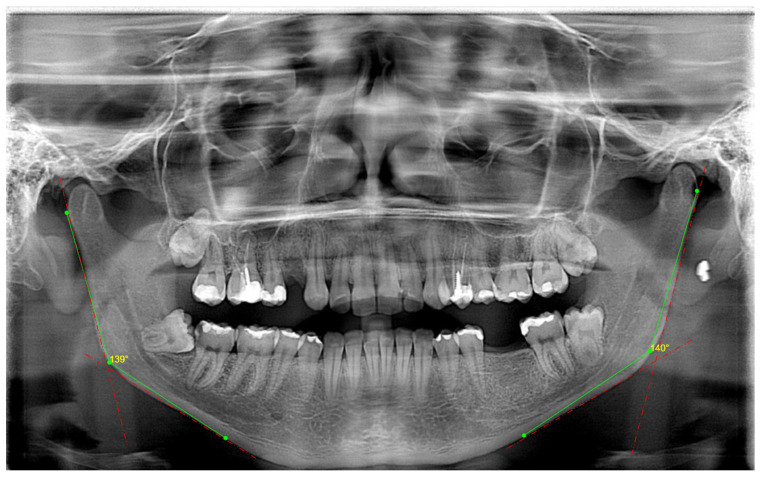
The WebCeph digital program—two lines were drawn: one tangent to the lower border of the mandible and the other to the distal border of the ascending ramus and the condyle on either side.

In Figure 5, we present the angular measurements exactly as we performed in this study in the WebCeph digital program; we measured the gonial angles bilaterally, on both sides, the results being very similar to the results we obtained by performing these measurements on the lateral cephalograms.

In order to measure, a line tangent to the mandibular lower border and another line tangent to the distal border of the ascending ramus and the condyle on either side were drawn.

## 3. Results

Our sample, comprising 128 individuals, provides an analysis of several mandibular and craniofacial parameters with a balanced gender distribution, with 52% female (n = 67) and 48% male (n = 61) participants. The median age of the cohort is 21 years, with an interquartile range (IQR) of 16 to 29 years, indicating a relatively young study population.

The functional mandibular angle (FMA) exhibits a median value of 22, with an IQR of 17 to 25, reflecting the range of mandibular growth and development among the participants. The gonial angle assessed using cephalometric methods presents a median of 121 degrees (IQR: 116–127), which suggests a consistent anatomical configuration across the cohort. Measurements of the mandibular ramus height using cephalometry imaging reveal a median value of 44 mm (IQR: 41–48 mm), indicating minimal variation in vertical ramus dimensions. The mandibular body length assessed via cephalometric methods has a median value of 70 mm, with an IQR of 65 to 76 mm, demonstrating uniformity in horizontal mandibular development. Orthopantomography (OPG) assessments of the gonial angle on the right and left sides yield medians of 121 degrees (IQR: 116–127) and 122 degrees (IQR: 117–127), respectively, confirming symmetry in angular measurements. Similarly, mandibular ramus height measurements using OPG on the right and left sides show medians of 44.0 mm (IQR: 40.0–47.0 mm) and 43 mm (IQR: 40–48 mm), with negligible side-to-side differences.

The mandibular body width measured through OPG is consistent across the sides, with the right side presenting a median of 71 mm (IQR: 67–76 mm) and the left side having a median of 71 mm (IQR: 67–75 mm). These findings underscore the symmetrical and proportional mandibular architecture within the study population (Table 2).

Table 3 presents a comparison of craniofacial measurements between female and male participants, analyzing differences across variables such as age, gonial angles, the mandibular ramus height, and mandibular body dimensions. A total of 128 participants were included, with 67 females and 61 males.

The median age of the male group (23.0 years; IQR: 15.7–30.7) was slightly higher than that of the female group (21.0 years; IQR: 17.0–26.0), but this difference was not statistically significant (*p* = 0.42). Similarly, the FMA angle showed comparable distributions between females (median: 21.0°; IQR: 16.0–25.0) and males (median: 22.0°; IQR: 18.7–26.0), with no significant difference (*p* = 0.50).

Significant differences were observed in gonial angles measured on lateral cephalograms and panoramic radiographs. The Gonial Angle (lateral ceph) was higher in males (median: 123.4°; IQR: 118.5–128.9) compared to females (median: 120.4°; IQR: 115.4–125.2), with a significant *p*-value of 0.01. The gonial angle on the right side measured on OPG was also larger in males (median: 123.0°; IQR: 117.0–129.0) than in females (median: 120.0°; IQR: 114.9–125.8), with *p* = 0.01. However, no significant difference was noted for the left gonial angle (*p* = 0.20).

Mandibular ramus height measurements revealed consistent differences, with males exhibiting significantly greater heights than females across all methods and sides. For instance, the mandibular ramus height (lateral ceph) was 46.9 mm (IQR: 42.0–52.1) in males, compared to 41.9 mm (IQR: 38.5–45.0) in females (*p* < 0.01). The same trend was observed in right and left ramus heights measured on OPG, with males showing significantly higher values (*p* < 0.01 for both).

Mandibular body dimensions also demonstrated significant sex differences. Males had longer mandibular bodies measured on lateral cephalometric radiographs (median: 73.0 mm; IQR: 66.7–77.3) compared to females (median: 69.8 mm; IQR: 64.7–73.9), with *p* = 0.01. Similarly, mandibular body widths on both the right and left sides measured on OPG were larger in males than females, with significant *p*-values of <0.01 and 0.02, respectively.

**Figure 6:**
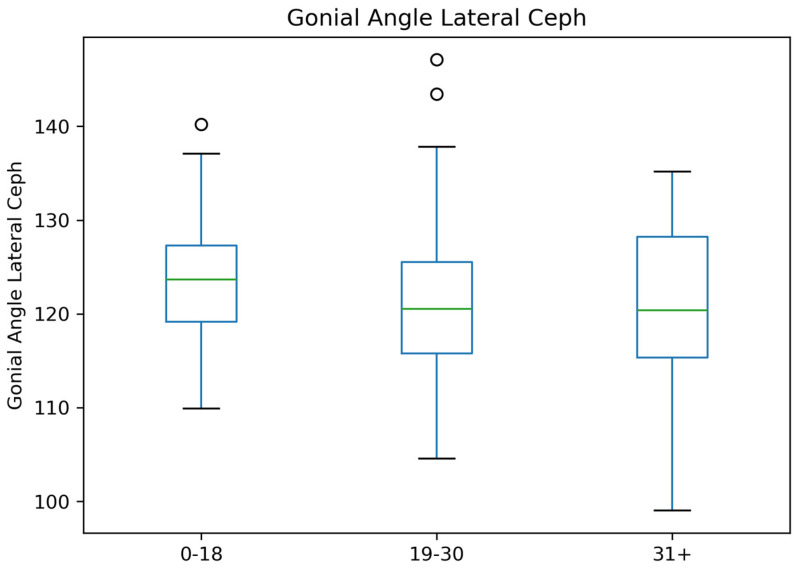
Age-Wise Comparison of Gonial Angle Lateral Ceph.

For the gonial angle on Lateral Ceph, the median gonial angle remains consistent across all age groups. The variability is moderate, and the whiskers show no extreme deviation from the central tendency. Some outliers are observed in the 0–18 and 19–30 groups (Figure 6).

**Figure 7:**
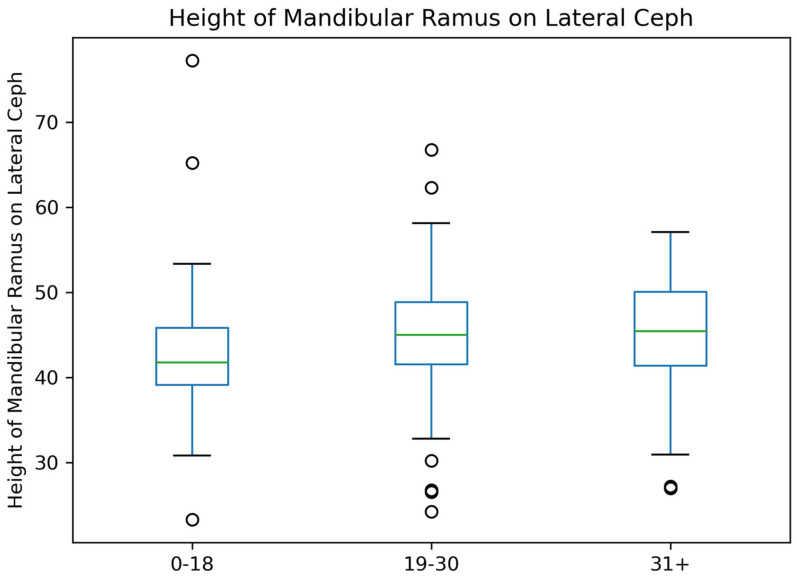
Comparison of Mandibular Ramus Height Across Age Groups.

For the height of the mandibular ramus on Lateral Ceph, the median height remains consistent across the age groups (0–18, 19–30, 31+). The variability within each group, as represented by the interquartile range, is relatively small. A few outliers are observed, particularly in the younger (0–18) and middle (19–30) age groups (Figure 7).

**Figure 8:**
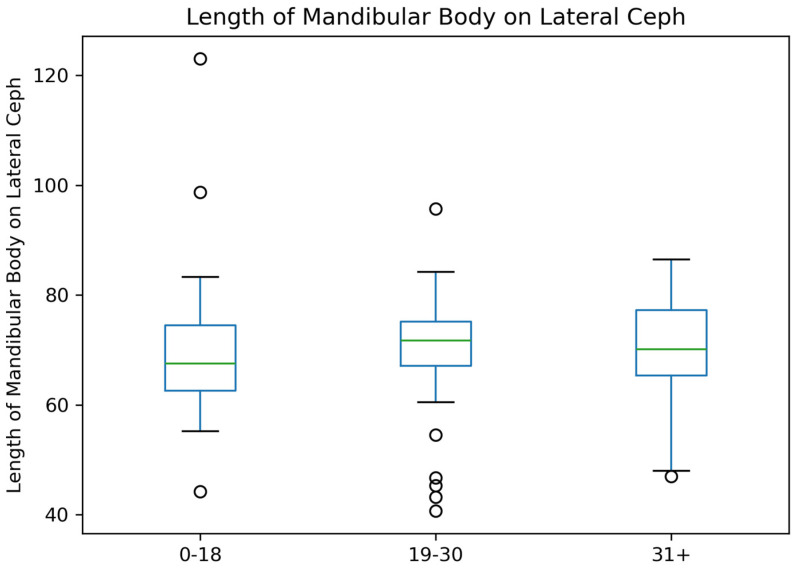
Mandibular Body Length Distribution by Age Groups.

For the length of the mandibular body on Lateral Ceph, the median length appears stable across the three age groups. The 19–30 age group shows a slightly wider interquartile range compared to the others. Outliers are present in all age groups, with a higher concentration in the 19–30 group (Figure 8).

Table 4 presents the correlation matrix for mandibular ramus height measurements from Lateral Ceph and orthopantomography (OPG) methods. Spearman’s correlation coefficient indicates the strength of the monotonic relationship between the variables. High positive correlations were found between the measurements. For instance, the correlation between Ramus Height Lateral Ceph and Ramus Height Right OPG was 0.901, with a 95% confidence interval ranging from 0.863 to 0.930, and the relationship was statistically significant (*p* < 0.001). Similarly, the correlation between Ramus Height Lateral Ceph and Ramus Height Left OPG was 0.844, also statistically significant (*p* < 0.001). The correlation between the right and left OPG ramus height measurements was 0.800, with a confidence interval of 0.725 to 0.855, and was also statistically significant (*p* < 0.001). The high correlations suggest strong consistency and agreement between the cephalometry and OPG methods for measuring the mandibular ramus height.

Table 5 summarizes the correlation between gonial angle measurements using cephalometric imaging (lateral ceph) and orthopantomography or panoramic radiograph (OPG) methods (right and left). Spearman’s rho (rs) was used to evaluate monotonic relationships, and the results are presented with degrees of freedom and significance levels.

Strong positive correlations were observed between Gonial Angle Lateral Ceph and both OPG measurements. For instance, the correlation between Gonial Angle Lateral Ceph and Gonial Angle Right OPG was 0.946 (*p* < 0.001), indicating a near-perfect monotonic relationship. Similarly, the correlation between Gonial Angle Lateral Ceph and Gonial Angle Left OPG was 0.858 (*p* < 0.001). The correlation between the right and left OPG measurements was also strong, with rs = 0.811 (*p* < 0.001).

Males demonstrated consistently larger craniofacial measurements, including gonial angles, ramus heights, and mandibular body dimensions, compared to females. These findings highlight the anatomical differences between sexes, which are critical for individualized orthodontic and surgical planning. Variables such as age and the FMA angle did not show significant differences between the sexes, suggesting that other factors primarily drive the observed differences in craniofacial measurements.

## 4. Discussion

This study aimed to evaluate the associations of digital orthopantomography (OPG) or panoramic radiography in determining angular and linear craniofacial measurements compared to lateral cephalograms, focusing on their applicability in diagnosing facial asymmetry and skeletal patterns. The findings demonstrated that while angular measurements on OPGs, such as the gonial angle, exhibit strong correlations with lateral cephalometric counterparts, linear horizontal measurements like the mandibular body length showed weaker correlations.

The gonial angle measurements on OPGs showed strong positive correlations with lateral cephalograms (rs = 0.946, *p* < 0.001 for the right side; rs = 0.858, *p* < 0.001 for the left side). These results align with prior research by Shahabi et al. [2] and Larheim and Svanaes [28], confirming the reliability of OPGs for angular assessments. This consistency highlights the utility of OPGs in evaluating angular relationships of the mandible, especially in diagnosing facial asymmetry. Similarly, the mandibular ramus height measurements on OPGs also correlated strongly with lateral cephalograms (rs = 0.901, *p* < 0.001), supporting the findings of Ongkosuwito et al. [29] that vertical measurements on OPGs are generally accurate under optimal conditions.

However, the mandibular body length showed weaker correlations between the two modalities, emphasizing the limitations of OPGs for horizontal measurements. This discrepancy can be attributed to the inherent magnification and distortion in panoramic imaging, as noted by Tronje et al. [30], where horizontal dimensions are particularly susceptible to non-linear distortions.

Our findings align with earlier studies suggesting that panoramic radiographs can be a reliable adjunct for angular and vertical measurements, provided the patient is appropriately positioned. For instance, Mattila et al. [31] reported that gonial angles on OPGs closely matched those measured on dry skulls. Conversely, studies like those by Nohadani and Ruf [32] highlighted the limitations of OPGs for the longitudinal evaluation of vertical facial dimensions, consistent with our results for the mandibular body length.

While Akcam et al. [33] and Kurt et al. [34] emphasized the utility of OPGs for assessing skeletal patterns and mandibular asymmetry, they also cautioned against over-reliance on OPGs for precise quantitative assessments of horizontal parameters. This study corroborates these findings, particularly for the mandibular body length, which exhibited weaker agreement between the two imaging modalities.

The strong correlation between angular measurements on OPGs and lateral cephalograms underscores the potential of OPGs as a preliminary diagnostic tool in orthodontics. OPGs provide the advantage of visualizing the right and left sides separately without the superimposition challenges inherent in lateral cephalograms. This makes them particularly useful in assessing facial asymmetry and angular relationships in clinical practice.

However, caution is warranted when interpreting linear measurements from OPGs. As noted, the prediction percentages for horizontal and vertical measurements remain low due to nonlinear distortion and inconsistent magnification factors [14,30]. Thus, while OPGs are valuable for initial screening, lateral cephalograms remain the gold standard for precise cephalometric analysis.

Slagsvold and Pedersen [24] highlight the inherent limitations of lateral cephalograms in accurately depicting angular measurements unless the plane of the angle is parallel to the film. Given that the gonial angle recorded on a lateral cephalogram represents an intermediate value between the left and right gonial angles, it inherently reflects potential deformations on either side. This geometric limitation underscores the need for alternative imaging modalities, such as orthopantomograms (OPGs), particularly for assessing mandibular asymmetry.

This study utilized three types of radiographs—panoramic radiographs, posteroanterior radiographs, and lateral cephalometric radiographs—to evaluate the craniofacial morphology pre- and post-treatment. The findings from the panoramic radiographs taken after therapy showed no signs of root resorption, while the upper and lower incisors exhibited proclination. These observations align with prior studies indicating that orthodontic interventions influence incisor positioning, particularly in patients with skeletal discrepancies.

Addressing facial asymmetry remains a significant clinical challenge, particularly in cases of severe mandibular deviation. For non-growing patients, a combination of orthodontic and surgical interventions is often the preferred approach. However, previous studies have reported cases where the spontaneous recovery of the condyles occurs, especially in pediatric populations, where misdiagnosed condylar fractures may contribute to asymmetry [35]. Asymmetries can be classified into three categories—functional, skeletal, and dental—each requiring distinct diagnostic and therapeutic considerations. Functional asymmetries often result from occlusal interferences, such as a restricted upper arch or a malpositioned tooth deflecting the mandible during closure. In contrast, skeletal asymmetries may involve the mandible, maxilla, or both, whereas dental asymmetries can arise from factors such as early deciduous tooth loss or parafunctional habits like thumb sucking. Our findings reinforce the importance of early intervention in cases of crossbite-related asymmetries to mitigate long-term functional and esthetic complications [35].

This study further supports the role of OPGs as a reliable tool for gonial angle measurement, providing precision comparable to that of lateral cephalograms, particularly in Skeletal Class I and III patients. However, for Skeletal Class II cases, OPGs demonstrated superior accuracy in distinguishing right and left gonial angles, as they eliminate the superimposition artifacts present in lateral cephalograms [36]. This is particularly relevant when assessing mandibular asymmetry, as OPGs allow for the independent analysis of each side of the mandible. Furthermore, no significant differences were observed between male and female subjects in terms of gonial angle measurements across various malocclusion groups, consistent with previous findings [36].

The reliability of panoramic radiography in measuring the gonial angle and ramus height has been corroborated in previous studies [37]. A comparative analysis of panoramic and cephalometric radiographs demonstrated that the mean gonial angle was 127.07 ± 6.10° on OPGs and 127.5 ± 6.67° on lateral cephalograms, with no statistically significant difference between the two methods. Additionally, there was no significant discrepancy between right and left gonial angles [38]. These findings further validate the use of OPGs in angular assessments, supporting their clinical utility in orthodontic diagnostics.

Our results demonstrate strong correlations between the angular measurements on OPGs and lateral cephalograms, particularly for the gonial angle and vertical linear parameters such as the mandibular ramus height. However, horizontal linear measurements, such as the mandibular body length, exhibited weaker correlations between the two imaging modalities. This discrepancy can be attributed to the inherent magnification and distortion in panoramic imaging, which disproportionately affect the horizontal dimensions [30]. The non-linear distortion in OPGs limits their reliability for precise horizontal measurements, necessitating caution when using these radiographs for evaluating the mandibular body length.

These findings reinforce the value of OPGs as an effective adjunct to lateral cephalograms in orthodontic assessments. While OPGs provide accurate and reliable angular and vertical measurements, their limitations in horizontal dimension analysis highlight the continued importance of cephalometric imaging for comprehensive skeletal evaluations. Future research should focus on refining imaging protocols to minimize distortion in panoramic radiography and explore the integration of advanced imaging technologies, such as cone–beam-computed tomography (CBCT), to enhance the diagnostic precision.

## 5. Limitations and Future Directions

Despite the promising findings, this study has several limitations. First, the sample size, while robust, was restricted to Romanian patients, potentially limiting the generalizability of the results. Second, variability in patient positioning during OPG acquisition, despite standardized protocols, may have influenced the accuracy of certain measurements. Another limitation would be, for example, potential confounding variables such as occlusal differences, temporomandibular joint problems, and prior dental treatments not being taken into consideration in this study. Additionally, this study focused solely on two-dimensional imaging modalities, excluding emerging three-dimensional technologies like cone–beam-computed tomography (CBCT), which offer superior accuracy for craniofacial assessments.

AI has the ability to completely transform medicine, especially in the areas of diagnostic imaging, which includes orthodontics. One of the fields which has most benefited from the introduction of AI technology is orthodontics, as the ongoing development of AI algorithms that support pretreatment diagnostic processes enables the visualization of results and streamlines decision making during treatment. But, because AI is so complex and unpredictable, these tools should be used carefully, and their output should be manually verified on a frequent basis. Orthodontics is one of the many medical specializations that have changed as a result of artificial intelligence (AI) in medicine. AI has demonstrated encouraging results in improving the precision of diagnosis, treatment planning, and outcome prediction. With the advent of numerous AI tools and applications, their use in orthodontic clinics around the world has grown [39]. In a number of medical specializations, AI algorithms have already demonstrated efficacy, outperforming skilled practitioners [40,41,42,43,44].

Future research should aim to standardize OPG acquisition protocols further, minimizing variability caused by patient positioning and equipment settings. Larger, multicenter studies involving diverse populations are needed to establish normative values for panoramic metrics and validate their consistency across different imaging systems. Additionally, integrating artificial intelligence algorithms to enhance the landmark detection and measurement accuracy on OPGs may help address current limitations [15,21]. Comparative studies involving CBCT should also be conducted to explore its potential as a complementary or alternative diagnostic tool to OPGs and lateral cephalograms.

## 6. Conclusions

This study highlights the utility of OPGs for angular and vertical mandibular measurements, offering a reliable, non-invasive, and cost-effective option for initial orthodontic assessments. However, their limitations for horizontal measurements necessitate careful interpretation and the complementary use of lateral cephalograms when precision is paramount. Standardizing OPG protocols and leveraging advancements in imaging technologies are essential steps toward enhancing their diagnostic potential. While OPGs cannot replace lateral cephalograms entirely, they remain a valuable adjunct in orthodontic diagnostics, particularly for assessing asymmetry and angular parameters, but the use of panoramic radiography has the advantage of simplifying the measurement of the gonial angles compared to the lateral ceph because, in some rarer cases, the anatomical landmarks can superimpose the latter. As precise as a lateral cephalogram is, panoramic radiography can be used to measure the gonial angle and ramus height.

## Figures and Tables

**Table 1:** Measurement and landmark list.

S. No.	Measurement	Description
1	ANB (°)	Angle between the Nasion-A point and the Nasion-B point plane [23].
2	FMA (°)	Angle formed by the FH plane and the mandibular plane [23].
3	GONIAL ANGLE (°)	At lateral cephalograms, it was determined at the junction of the mandibular and ramus planes. A line tangent to the lower border of the mandible and another line tangent to the distal border of the ascending ramus and the condyle on either side were drawn in order to measure the gonial angle in the panoramic radiographs [24]. The mandibular plane and the ramus plane are built point of junction.
4	RAMUS HEIGHT (mm)	The distance between Ag and Snp [25].
5	MANDIBULAR BODY LENGTH (mm)	The distance between point Go and point M [25].

FH plane—Frankfort plane, Ag—antegonion, Snp—sigmoid notch point, Go—Gonion, M—mandibular midpoint.

**Table 2:** Patients’ baseline characteristics.

Characteristic	N = 128
Sex	
Female (F)	67 (52%)
Male (M)	61 (48%)
Age	21 (16, 29)
FMA	22 (17, 25)
Gonial Angle (Lateral Ceph)	121 (116, 127)
Mandibular Ramus Height (Lateral Ceph)	44 (41, 48)
Mandibular Body Length (Lateral Ceph)	70 (65, 76)
Gonial Angle Right (OPG)	121 (116, 127)
Gonial Angle Left (OPG)	122 (117, 127)
Mandibular Ramus Height Right (OPG)	44.0 (40.0, 47.0)
Mandibular Ramus Height Left (OPG)	43 (40, 48)
Mandibular Body Width Right (OPG)	71 (67, 76)
Mandibular Body Width Left (OPG)	71 (67, 75)

**Table 3:** Comparison of Craniofacial Measurements Between Female and Male Participants.

Variable	N	Female (F) (N = 67) (Median, Q1, Q3)	Male (M) (N = 61) (Median, Q1, Q3)	*p*-Value
Age (years)	128	21.0 (17.0, 26.0)	23.0 (15.7, 30.7)	0.42
FMA (°)	121	21.0 (16.0, 25.0)	22.0 (18.7, 26.0)	0.5
Gonial Angle (Lateral Ceph) (°)	128	120.4 (115.4, 125.2)	123.4 (118.5, 128.9)	0.01
Mandibular Ramus Height (Lateral Ceph) (mm)	128	41.9 (38.5, 45.0)	46.9 (42.0, 52.1)	<0.01
Mandibular Body Length (Lateral Ceph) (mm)	128	69.8 (64.7, 73.9)	73.0 (66.7, 77.3)	0.01
Gonial Angle Right (OPG) (°)	128	120.0 (114.9, 125.8)	123.0 (117.0, 129.0)	0.01
Gonial Angle Left (OPG) (°)	128	123.0 (116.2, 125.0)	122.0 (117.0, 129.0)	0.2
Mandibular Ramus Height Right (OPG) (mm)	126	42.0 (39.0, 45.0)	46.0 (42.7, 50.0)	<0.01
Mandibular Ramus Height Left (OPG) (mm)	126	42.0 (38.0, 44.3)	47.0 (42.0, 51.0)	<0.01
Mandibular Body Width Right (OPG) (mm)	128	69.5 (66.3, 74.0)	73.7 (69.1, 77.2)	<0.01
Mandibular Body Width Left (OPG) (mm)	127	70.0 (66.0, 74.1)	72.0 (69.0, 76.3)	0.02

Data are presented as median (Q1, Q3). Measurements are in degrees (°) for angular values and millimeters (mm) for linear dimensions. Statistical significance was determined using the Wilcoxon test.

**Table 4:** Correlation Matrix for Mandibular Ramus Heights.

Variable	Ramus Height Lateral Ceph	Ramus Height Right OPG	Ramus Height Left OPG
Ramus Height Lateral Ceph	—		
Spearman’s rho	—	0.901 (95% CI: 0.863, 0.930)	0.844 (95% CI: 0.785, 0.888)
*p*-value	—	<0.001 ***	<0.001 ***
Ramus Height Right OPG		—	
Spearman’s rho		—	0.800 (95% CI: 0.725, 0.855)
*p*-value		—	<0.001 ***
Ramus Height Left OPG			—

Note: *** *p* < 0.001.

**Table 5:** Correlation Matrix for Gonial Angles.

Variable	Gonial Angle Lateral Ceph	Gonial Angle Right OPG	Gonial Angle Left OPG
Gonial Angle (Lateral Ceph)	—		
Spearman’s rho	—	0.946 ***	0.858 ***
Degrees of Freedom (DF)	—	126	126
*p*-value	—	<0.001	<0.001
Gonial Angle Right (OPG)		—	
Spearman’s rho		—	0.811 ***
Degrees of Freedom (df)		—	126
*p*-value		—	<0.001
Gonial Angle Left (OPG)			—

Note: *** *p* < 0.001.

## Data Availability

All data regarding this manuscript can be requested from the corresponding author at alexandru.motofelea@umft.ro.
